# Supplementary material for: Oxidative Stress Is Associated with Overgrowth in Drosophila l(3)mbt Mutant Imaginal Discs
Source: Cells. 2022 Aug 16;11(16):2542. doi: 10.3390/cells11162542 (PMC9406541; doi:10.3390/cells11162542)
Supplement: Supplementary file 1 [file cells-11-02542-s001.zip › cells-1848236-Figure S1.pdf]

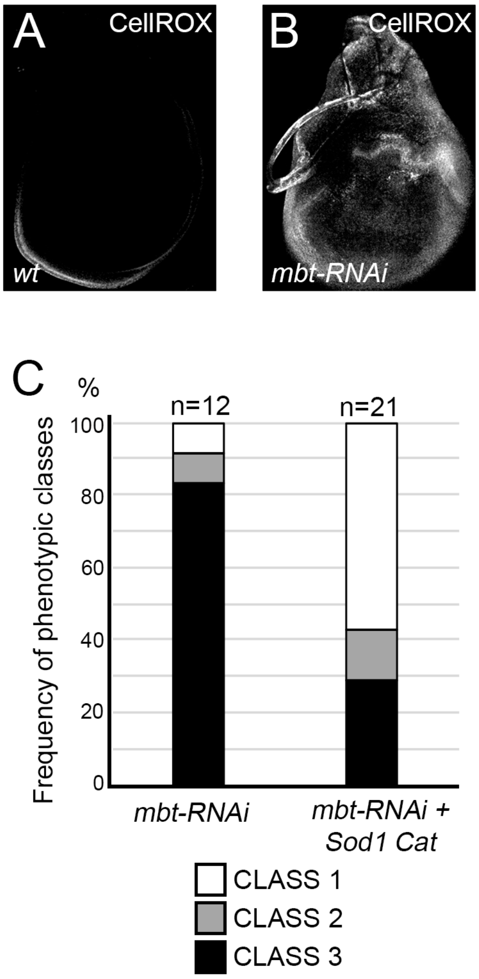

Figure S1. Oxidative stress detected with CellROX Deep Red and effect of the ROS scavengers Sod1 and Cat in *mbt-RNAi* mutant wing imaginal discs.
